# Individualized evaluation of risk and prognosis in uterine leiomyosarcoma patients with synchronous distant metastases: a real-world retrospective study

**DOI:** 10.3389/fonc.2024.1417226

**Published:** 2024-09-25

**Authors:** Zhongli Liu, Feng Gao, Tao Min, Qianqian Shang, Bin Wang, Jing Pu

**Affiliations:** ^1^ Day Treatment Center, Mianyang Central Hospital, Mianyang, Sichuan, China; ^2^ Department of Medical Oncology, Mianyang Central Hospital, Mianyang, Sichuan, China; ^3^ Department of Neurology, Mianyang Central Hospital, Mianyang, Sichuan, China; ^4^ Medical Oncology, Cancer Center, West China Hospital, Sichuan University, Chengdu, Sichuan, China; ^5^ Department of Internal Medicine, Mianyang Central Hospital, Mianyang, Sichuan, China

**Keywords:** uterine leiomyosarcoma, nomogram, distant metastases, risk factors, prognosis

## Abstract

**Background:**

Uterine leiomyosarcoma (uLMS) accounts for roughly 70% of all uterine sarcomas, with recurrence and mortality rates notably higher than those of other uterine tumors. The prognosis of uLMS patients who have distant metastases remains poor. The objective of this study was to determine independent risk variables related to distant metastases in patients with uLMS and prognostic factors for those with distant metastases. Subsequently, two practical nomograms were developed and validated to assess the probability of distant metastases and predict survival outcomes for these with distant metastases, respectively.

**Methods:**

A real-world retrospective study was carried out using data from patients diagnosed with primary uLMS in the Surveillance, Epidemiology, and End Results (SEER) database spanning the years 2010 to 2015. Univariate and multivariate logistic regression analyses were utilized to identify clinicopathological characteristics related to the risk of distant metastases, while univariate and multivariate Cox regressions were employed to determine prognostic factors. Then, a risk nomogram incorporating independent risk variables and a prognostic nomogram integrating independent prognostic factors were established in the training cohort and validated for accuracy in the validation cohort, respectively. Receiver operating characteristic (ROC) curves, area under the curve (AUC), and calibration curves were utilized to measure the accuracy of nomograms, while decision curve analysis (DCA) curves were employed to assess their clinical benefit capacity. Based on the median total point derived from the prognostic nomogram, patients were stratified into high- and low-risk groups. The differentiation ability of the prognostic nomogram was evaluated using Kaplan-Meier survival analysis with the log-rank test.

**Results:**

The study encompassed 1,362 patients diagnosed with uLMS, among whom 337 cases (24.7%) manifested synchronous distant metastases at the initial diagnosis. Univariate and multivariate logistic regression analyses identified race, histological grade, T stage, N stage, tumor size, surgery, and chemotherapy as independent risk factors for distant metastases in uLMS patients. The outcomes of both univariate and multivariate Cox analyses indicated that surgery and chemotherapy emerged as independent protective factors for prognosis in uLMS patients with distant metastases, whereas higher histological grade and T stage were identified as independent risk factors. The risk nomogram incorporating independent risk variables and the prognostic nomogram integrating independent prognostic factors could respectively predict the risk of metastases and the prognosis very effectively in both training and validation cohorts.

**Conclusions:**

In summary, we developed the novel well-validated risk nomogram to precisely assess the probability of metastases in uLMS patients and prognostic nomogram to predict the prognosis of those with distant metastases, providing decision-making guidance for tailoring individualized clinical management of these patients.

## Introduction

Leiomyosarcoma (LMS) is a rare and aggressive mesenchymal neoplasm characterized by smooth muscle differentiation, represents 10-20% of all newly diagnosed soft tissue sarcomas (STSs), which most frequently occurs in the extremities, retroperitoneum, or uterus (1). Uterine LMS (uLMS) is a particularly rare and aggressive subtype of LMSthat arises from the smooth muscle of the myometrium (2). The incidence of uLMS is around 0.55 cases per 100, 000 females, with the average age at diagnosis of 51 years old and the majority being in the perimenopausal stage (3). While uLMS accounts for just around 1% of all uterine malignancies, it covers approximately 70% of all uterine sarcomas and a significant proportion of fatalities from uterine malignancy (4). Compared to other types of uterine cancers, uLMS have a higher rate of recurrence and mortality (5).

Despite the majority of patients (60%) with uLMS being diagnosed at an early stage, prognosis for uLMS remains poor, with five-year survival rates ranging from 25% to 76% (6). Metastatic/Recurrent rates of uLMS ranged from 45% to 75% (7). The lung was the most common site of metastasis for uLMS, with bone, intra/extracranial, skin and soft tissue metastases also being relatively common (8). For patients with metastasis at initial diagnosis, the five-year survival rate dropped to about 10% to 15%, with mortality typically occurring within 2 years, and the median survival for stage IV patients was estimated to be about 12 months (9). For patients with metastatic or unresectable disease, the principles of management of other soft tissue sarcomas are followed. Chemotherapeutic agents with efficacy include doxorubicin, combination gemcitabine and docetaxel, or trabectedin and to a lesser extent dacarbazine or eribulin (10–12). To date, although various combinations or the addition of molecular-targeting agents to these chemotherapy backbones have surpassed doxorubicin therapy in terms of overall response rate (ORR) or progression-free survival (PFS), an improvement in OS is yet to be observed (11). Due to its rarity, there is currently still a paucity in literature surrounding subsequent lines of immunotherapy for uLMS. Although two case studies involving patients with metastatic uLMS reported dramatic reductions in tumour burden and aided prolonged disease stabilization with immune checkpoint inhibitor therapy (13, 14), clinical trials revealed neither single agent nivolumab or pembrolizumab have as yet failed to show any benefit or response in advanced uLMS patients (15, 16). Age at diagnosis, tumor size, tumor grade, presence of cervical invasion, tumor mitotic rate, locoregional metastases, and distant metastases have been identified as factors influencing the prognosis of uLMS patients (17). However, as far as we know, population-level estimates for the risk of distant metastases in uLMS patients are lacking, and there is a scarcity of studies providing reliable evidence regarding the associations between clinicopathological features and metastatic patterns. Therefore, early identification of synchronous distant metastases in uLMS patients and individualized survival prediction for those with distant metastases are pivotal for optimizing medical decision-making. In this study, independent risk variables related to distant metastases in patients with uLMS, and prognostic factors for those with distant metastases were identified based on the uLMS cohort in the Surveillance, Epidemiology, and End Results (SEER) database. Subsequently, the risk nomogram incorporating independent risk variables and the prognostic nomogram incorporating independent prognostic factors were established and verified to respectively predict the risk of metastases and the individualized prognosis. We hope that the application of these novel nomograms will provide guidance for clinical decision-making for uLMS patients.

## Materials and methods

### Study population

**Figure 1:**
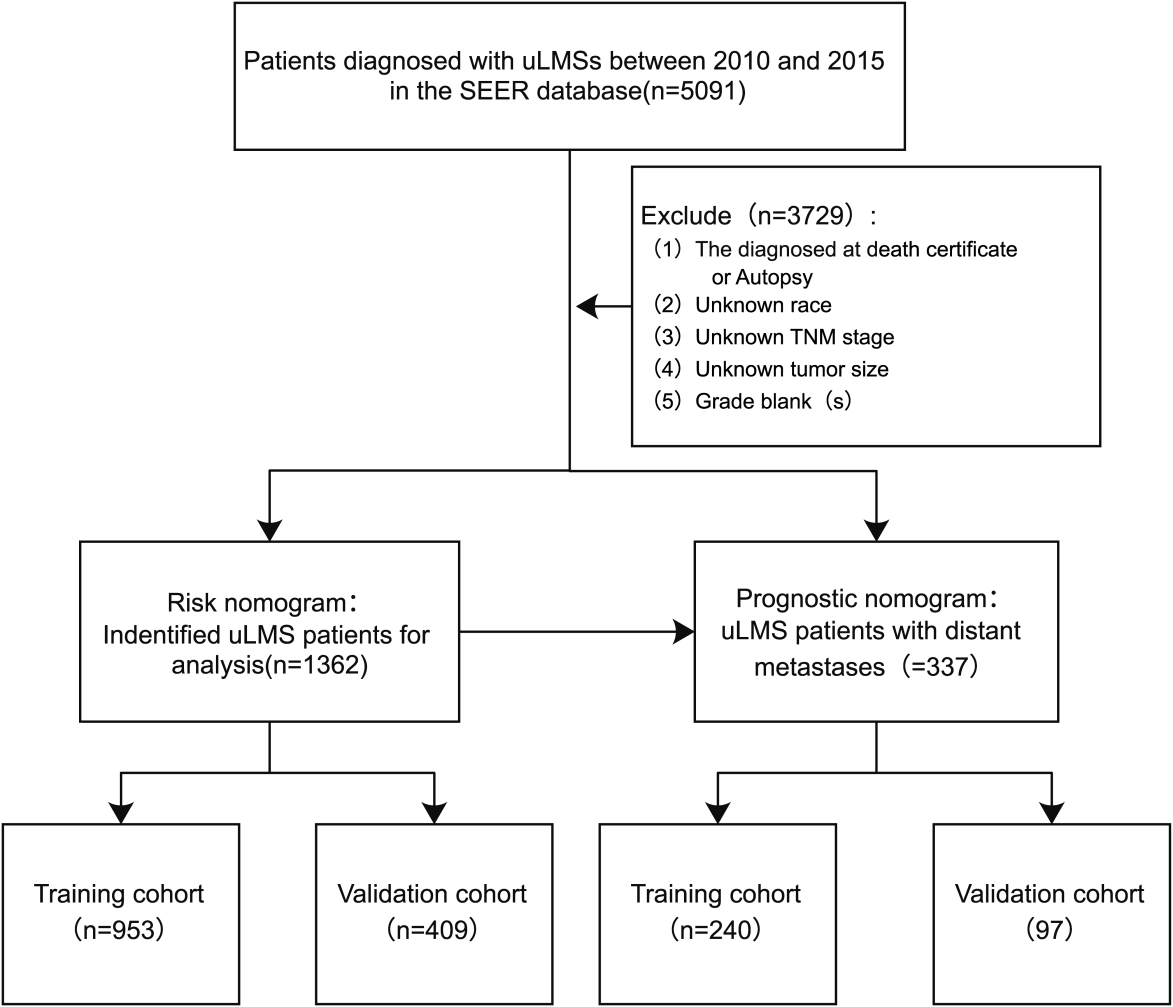
Flowchart of the screening procedure for patients with uLMS.

In this study, patient clinicopathological characteristics were extracted from the SEER database (https://seer.cancer.gov; accessed date March 1, 2024), covering all patients diagnosed with uLMS between 2010 and 2015. The SEER database is the national cancer registry of the United States, which consists of 18 population-based cancer registries among 14 states covering approximately 28% of the country’s population (18). Individuals diagnosed with uLMS coded 8896/3: Myxoid leiomyosarcoma, 8891/3: Epithelioid leiomyosarcoma, and 8890/3: Leiomyosarcoma NOS based on the International Classification of Diseases for Oncology (ICD-O-3) rules were identified from the SEER database. Those with uncertain survival times were excluded from the analysis. The flowchart of patient screening process were illustrated in **Figure 1**. Since the SEER database is a public database with anonymous patient information, our study does not require ethical approval and patients’ informed consent.

### Study variables

Clinicopathological characteristics of patients diagnosed with primary uLMS obtained from the SEER database encompassed race, age of diagnosis, tumor primary site, histological type, tumor size, AJCC stage, histological grade, surgery, chemotherapy, and radiotherapy. The age of diagnosis was categorized into three groups: ≤39, 40-59, and ≥60. And, the tumor size was categorized into three subgroups in terms of diameter: <50 mm, 50-100mm, and >100 mm. This study took OS as the primary endpoint.

### Statistical analyses

All statistical analyses were conducted using R software (version 4.2.1). All enrolled patients were randomly assigned to the training and validation cohorts at a 7:3 ratio. to determine differences in baseline characteristics between the training and validation cohorts, Chi-square (χ2) and Fisher’s exact tests were applied. Both univariate and multivariate logistic regression analyses were performed to screen clinicopathological variables correlated with the risk of distant metastases, and odds ratios (ORs) of variables were calculated and presented with 95% confidence intervals (CIs). For survival analysis of uLMS patients with distant metastases, univariate and multivariate Cox analyses were applied to screen prognostic factors. A risk nomogram incorporating independent risk variables and a prognostic nomogram integrating independent prognostic factors were established in the training cohort and validated in the validation cohort, respectively. The receiver operating characteristic (ROC) curves, area under the curve (AUC), and calibration curves were utilized to measure the accuracy of nomograms, while decision curve analysis (DCA) curves were employed to assess their clinical benefit capacity. Based on the median total point derived from the prognostic nomogram, patients were stratified into high- and low-risk groups. The differentiation ability of the prognostic nomogram was evaluated using Kaplan-Meier survival analysis with the log-rank test.

## Results

### Demographic and clinicopathological characteristics for uLMS

Following the inclusion and exclusion criteria, 1, 362 patients diagnosed with uLMS were included in this analysis and were randomly allocated to either the training cohort (n = 953) or the validation cohort (n = 409) at a ratio of 7:3. The detailed baseline clinicopathological characteristics of all included patients were shown in **Table 1**. Among all enrolled patients, 809 cases (59.4%) were 40-60 years old, and 941 cases (69.1%) were Caucasian with the remaining being Black or other. In terms of histological type, leiomyosarcoma, NOS constituted 91.3%, with the remaining cases being epithelioid leiomyosarcoma and myxoid leiomyosarcoma. In terms of histological grade, grade IV accounted for the largest proportion of known grades of differentiation. Regarding AJCC staging, 743 (54.6%) patients exhibited stage I tumors. As for TNM staging, the most prevalent T and N stages were T1 (65.9% in the training cohort and 68.0% in the validation cohort) and N0 (95.2% in the training cohort, and 95.1% in the validation cohort). Regarding treatment modalities, more than half of the uLMS patients had received surgery (95.0%) and chemotherapy (52.4%), but only a few (15.1%) had undergone radiotherapy. The results of the Chi-square test suggested no statistically significant differences in any of the covariates between the training and validation cohorts, indicating that the allocation was completely randomized.

**Table 1:** Baseline clinicopathological characteristics of uLMS patients.

	Training cohort (N=953,%)	validation cohort (N=409,%)	Overall (N=1362,%)	χ2	*P*
Age				0.947	0.623
≤39	60 (6.3)	28 (6.8)	88 (6.5)		
40-59	560(58.8)	249(60.9)	809(59.4)		
≥60	333(34.9)	132(32.3)	465(34.1)		
Race				2.286	0.319
Black	213(22.4)	85(20.8)	298(21.9)		
White	661(69.4)	280(68.5)	941(69.1)		
Other	79(8.3)	44(10.8)	123 (9.0)		
Histological type				0.354	0.838
Leiomyosarcoma, NOS	868(91.1)	375(91.7)	1243(91.3)		
Epithelioid leiomyosarcoma	54 (5.7)	20 (4.9)	74 (5.4)		
Myxoid leiomyosarcoma	31 (3.3)	14 (3.4)	45 (3.3)		
Histological grade				0.4552	0.797
I-II	82 (8.6)	39 (9.5)	121 (8.9)		
III-IV	418(43.9)	182(44.5)	600 (44.1)		
Unknown	453(47.5)	188(46.0)	641 (47.1)		
Tumor size				2.191	0.334
≤50	125(13.1)	64 (15.6)	189 (13.9)		
51-100	338(35.5)	132(32.3)	470 (34.5)		
≥100	490(51.4)	213(52.1)	703 (51.6)		
AJCC Stage				0.4712	0.925
Stage I	519(54.5)	224(54.8)	743 (54.6)		
Stage II	89 (9.3)	41 (10.0)	130 (9.5)		
Stage III	79 (8.3)	36 (8.8)	115 (8.4)		
Stage IV	266(27.9)	108(26.4)	374 (27.5)		
T Stage				3.119	0.374
T1	628(65.9)	278 (68.0)	906 (66.5)		
T2	153 (16.1)	67 (16.4)	220 (16.2)		
T3	112 (11.8)	48 (11.7)	160 (11.7)		
T4	60 (6.3)	16 (3.9)	76 (5.6%)		
N stage				<0.001	1.000
N0	907(95.2)	389 (95.1)	1296 (95.2)		
N1	46 (4.8)	20 (4.9)	66 (4.8)		
M stage				0.257	0.612
M0	713 (74.8)	312 (76.3)	1025 (75.3)		
M1	240(25.2)	97(23.7)	337 (24.7)		
Surgery				0.628	0.428
No	51 (5.4)	17 (4.2)	68 (5.0)		
Yes	902(94.6)	392(95.8)	1294(95.0)		
Radiotherapy				0.700	0.403
No	804(84.4)	353(86.3)	1157(84.9)		
Yes	149(15.6)	56(13.7)	205 (15.1)		
Chemotherapy				0.017	0.897
No/Unknown	455(47.7)	193(47.2)	648 (47.6)		
Yes	498(52.3)	216(52.8)	714 (52.4)		

### Risk variables of distant metastases in patients with uLMS

As shown in **Table 2**, out of the enrolled patients, 337 patients were diagnosed with distant metastases (stage M1) at initial diagnosis, representing 24.7% of the total cohort. Univariate and multivariate logistic regression analyses were conducted to identify clinicopathological variables correlated with the risk of distant metastases in uLMS patients. The univariate logistic analysis suggested that race, histological type, grade III-IV, tumor size≥100, T2-4 stages, N1 stage, surgery, and chemotherapy tended to be significantly associated with distant metastases. Subsequently, these variables were further incorporated into the multivariate logistic regression analysis, revealing that race, histological grade, T stage, N stage, tumor size, surgery, and chemotherapy were conclusively identified as independent risk factors for distant metastases in newly diagnosed uLMS patients (**Table 2**).

**Table 2:** Univariate and multivariate logistics regression analyses of distant metastases in patients with uLMS.

variables	Univariate analysis			Multivariate analysis		
	OR	95%CIs	P-value	OR	95%CIs	P-value
Age(year)			<0.001			
39	Reference					
40_59	1.33	0.85-2.15	0.317			
Age60	1.54	0.97-2.52	0.139			
Race						
Black	Reference					
White	0.52	0.41-0.656	<0.001	0.54	0.410-0.705	<0.001
Other	0.58	0.38-0.86	0.024	0.64	0.408-1.002	0.106
Histological type						
Leiomyosarcoma, NOS	Reference					
Epithelioid leiomyosarcoma	0.51	0.28-0.86	0.043	0.55	0.30-0.96	0.090
Myxoid leiomyosarcoma	0.73	0.38-1.32	0.406	0.83	0.41-1.61	0.662
Grade						
I-II	Reference					
III-IV	3.17	1.99-5.33	<0.001	2.07	1.26-3.55	0.021
Unknown	2.27	1.42-3.83	0.006	1.42	0.86-2.45	0.275
Size						
≤50	Reference					
≥100	2.70	1.89-3.95	<0.001	2.09	1.390-3.216	0.004
51-100	1.46	0.10-2.18	0.112	1.61	1.05-2.54	0.075
AJCC.Stage.						
Stage I	Reference					
Stage II	1.00	8.53 × 10^(-19)^ - 1.17 ×10^18^	1			
Stage III	1.00	9.00 × 10^(-20)^ - 1.11 ×10^19^	1			
Stage IV	2.12 x 10^10^	2.38 × 10^261^ - 2.02 ×10^247^	0.982			
T-Stage.						
T1	Reference					
T2	3.12	2.38-4.09	<0.001	2.24	1.66-3.02	<0.001
T3	3.06	2.25-4.14	<0.001	1.66	1.17-2.34	0.017
T4	5.23	3.49-7.85	<0.001	3.69	2.35-5.76	<0.001
N-stage						
N0	Reference					
N1	4.84	3.17-7.45	<0.001	2.34	1.45-3.79	0.004
Surgery						
No	Reference					
Yes	0.08	0.05-0.13	<0.001	0.089	0.05-0.15	<0.001
Radiation						
No	Reference					
Yes	1.14	0.85-1.50	0.453			
Chemotherapy						
No/Unknown	Reference					
Yes	3.04	2.43-3.82	<0.001	2.80	2.17-3.61	<0.001

### Development and validation of the risk nomogram to predict distant metastases

**Figure 2:**
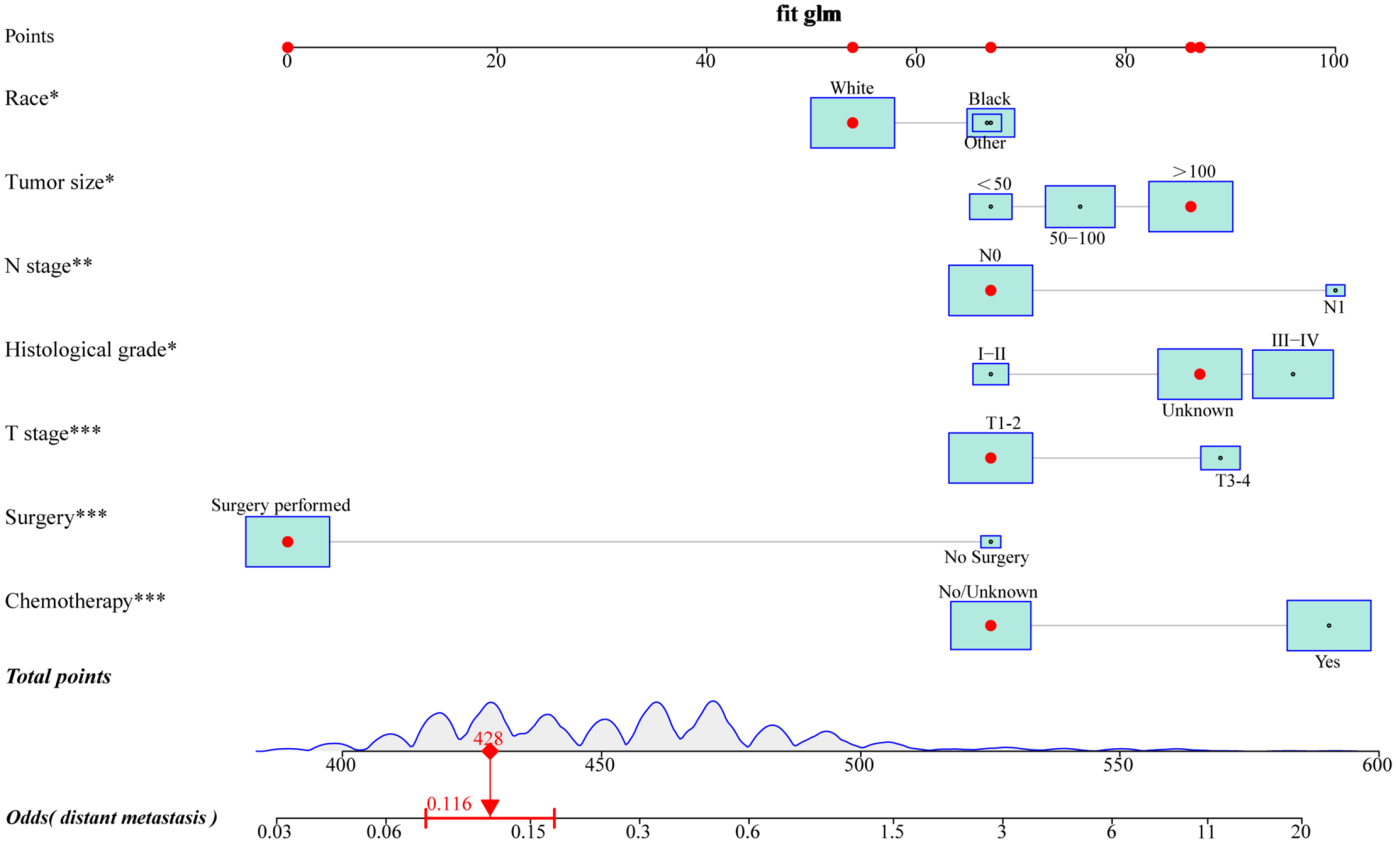
A novel risk nomogram was established to predict the probability of distant metastases in uLMS patients. based on seven independent risk factors, including race, histological grade, T stage, N stage, tumor size, surgery, and chemotherapy. The size of the box reflected the distribution of the variables, with a larger size indicating a higher proportion. Each level of these variables was assigned a specific point on the scale, and a total point could be obtained for the individual patients by summing each point form the above seven independent risk factors. A line was drawn vertically downward from the total points scale to the last axis to obtain the corresponding probability of distant metastases.

**Figure 3:**
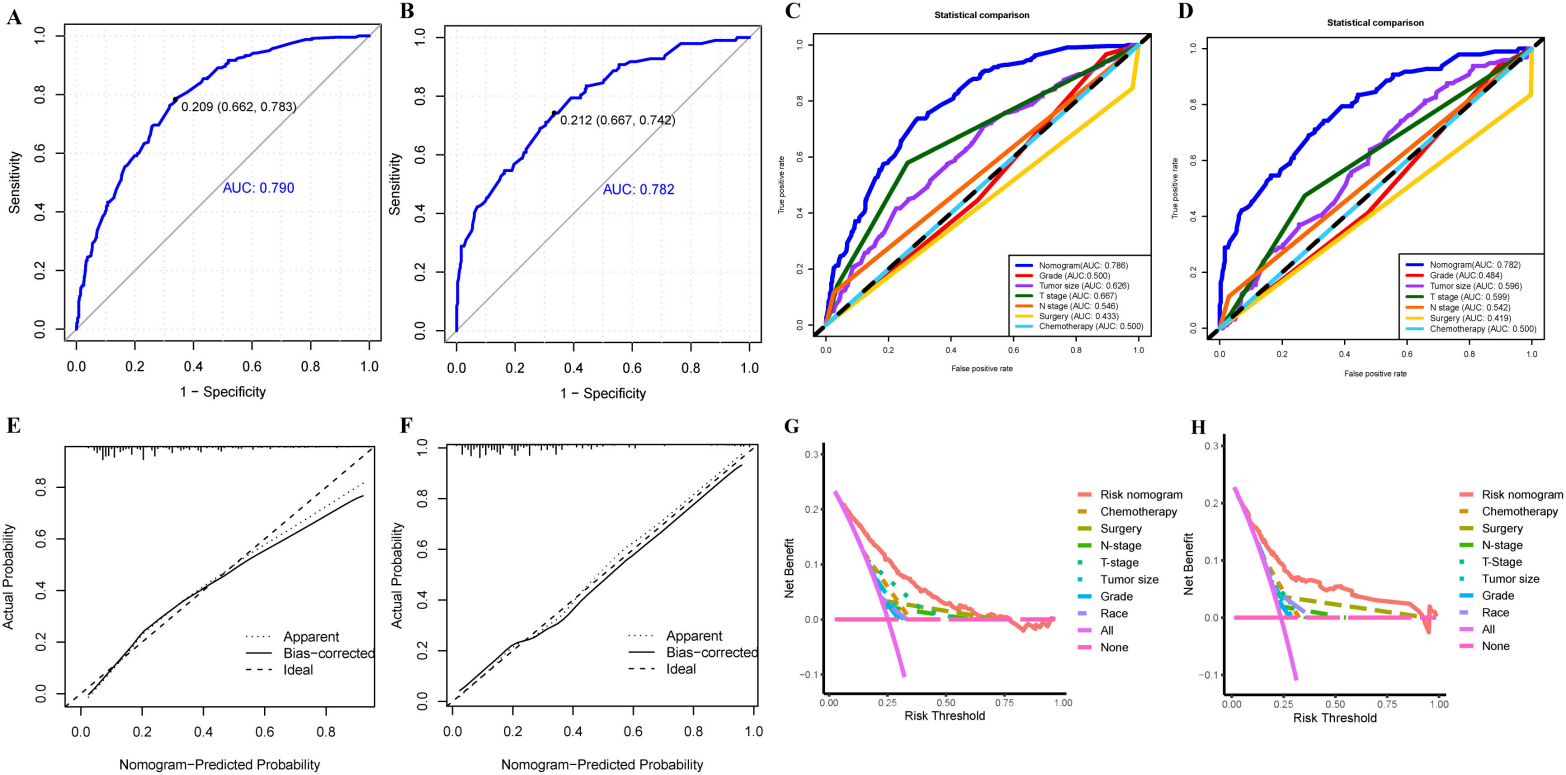
The ROC curve analysis of the risk nomogram in training **(A)** and validation **(B)** cohorts; The discrimination between the risk nomogram and independent risk factors (race, histological grade, T stage, N stage, tumor size, surgery, and chemotherapy) for predicting the probability of distant metastases in uLMS patients was compared by ROC curves in training **(C)** and validation **(D)** cohorts; **(E)** The nomogram’s calibration curve in the training **(E)** and validation **(F)** cohorts; Decision curves of the risk nomogram and independent risk factors in the training **(G)** and validation **(H)** cohorts.

To provide a more intuitive prediction of the risk of distant metastases in patients with uLMS, a novel risk nomogram was devised utilizing the seven independent risk predictors (**Figure 2**). The total score based on the calculation of each variable point was correlated with the probability of organ metastases. Then, ROC curves were established that exhibited excellent predictive ability, with AUCs of 0.772 and 0.776 for the nomogram in the training and validation cohorts, respectively (**Figures 3A, B**). Additionally, ROC curves for all independent predictors were generated (**Figures 3C, D**), illustrating the superior discriminative capacity of the risk nomogram in comparison to independent predictors across both training and validation cohorts. Of paramount importance, the calibration curves of the nomogram demonstrated excellent consistency between the observed and predicted outcomes in both the training and validation cohorts (**Figures 3E, F**). Finally, DCA curves indicated that the risk nomogram exhibited excellent performance compared with individual independent risk predictors, which could be utilized as a precise tool for clinical metastasis evaluation (**Figures 3G, H**).

### Analysis of prognostic factors for uLMS patients with distant metastases

In the current investigation, a total of 337 patients diagnosed with uLMS exhibited distant metastases, and they were randomly allocated into a training cohort and a validation cohort at a ratio of 7:3 (**Table 3**). The Chi-square test revealed no significant discrepancies in any variables between the two cohorts. Subsequently, univariate Cox regression analysis was employed to identify robust prognostic factors, confirming five clinicopathological variables including race, pathological grade, T stage, surgery, and chemotherapy related to prognosis in uLMS patients with distant metastases. These variables were further subjected to multivariate Cox analysis, wherein the final results indicated that surgery and chemotherapy emerged as independent protective factors for the prognosis of uLMS patients with distant metastases (HR < 1, P < 0.05; **Table 4**). Conversely, grade III-IV and T3-4 stages were identified as independent risk factors for the prognosis (HR > 1, P < 0.05; **Table 4**). The Kaplan–Meier analysis was further performed according to the results of the multivariable analysis, which revealed obvious discrimination on prognosis stratified by single independent prognostic parameters (**Supplementary Figure S1**).

**Table 3:** Baseline clinical characteristics of uLMS patients with distant metastases.

	Training cohort (N=240, %)	validation cohort (N=97, %)	Overall (N=337, %)	χ2	*P*
Age				0.947	0.623
≤39	13 (5.4)	4 (4.1)	17 (5.0)		
40-59	135 (56.3)	60 (61.9)	195 (57.9)		
≥60	92 (38.3)	33 (34.0)	125 (37.1)		
Race				2.327	0.312
Black	71 (29.6)	33 (34.0)	104 (30.9)		
White	145 (60.4)	59 (60.8)	204 (60.5)		
Other	24 (10.0)	5 (5.2)	29 (8.6)		
Histological type				0.703	0.704
Leiomyosarcoma, NOS	225 (93.8)	92 (94.8)	317 (94.1)		
Epithelioid leiomyosarcoma	9 (3.8)	2 (2.1)	11 (3.3)		
Myxoid leiomyosarcoma	6 (2.5)	3 (3.1)	9 (2.7)		
Grade				0.704	0.465
I-II	8 (3.3)	6 (6.2)	14 (4.2)		
III-IV	125 (52.1)	51 (52.6)	176 (52.2)		
Unknown	107 (44.6)	40 (41.2)	147 (43.6)		
Size				0.916	0.633
≤50	21 (8.8)	6 (6.2)	27 (8.0)		
51-100	156(65.0)	62 (63.9)	218 (64.7)		
≥100	63 (26.3)	29 (29.9)	92 (27.3)		
T Stage				6.572	0.087
T1	101 (42.1)	51 (52.6)	152 (45.1)		
T2	61 (25.4)	24 (24.7)	85 (25.2)		
T3	44 (18.3)	17 (17.5)	61 (18.1)		
T4	34 (14.2)	5 (5.2)	39 (11.6)		
N stage				1.11E-30	1
N0	212 (88.3)	86 (88.7)	298 (88.4)		
N1	28 (11.7)	11 (11.3)	39 (11.6)		
Surgery				0.007	0.936
No	37 (15.4)	16 (16.5)	53 (15.7)		
Yes	203 (84.6)	81 (83.5)	284 (84.3)		
Radiotherapy				0.576	0.448
No	198 (82.5)	84 (86.6)	282 (83.7)		
Yes	42 (17.5)	13 (13.4)	55 (16.3)		
Chemotherapy				1.34E-29	1
No/Unknown	67 (27.9)	27 (27.8)	94 (27.9)		
Yes	173 (72.1)	70 (72.2)	243 (72.1)		

**Table 4:** Univariate and multivariate Cox analyses in uLMS patients with distant metastases.

variables	Univariate analysis			Multivariate analysis		
	HR	95%CIs	P-value	HR	95%CIs	P-value
Age(year)						
Age39	Reference					
Age40_59	1.07	0.61- 1.88	0.823			
Age60	1.40	0.79- 2.49	0.249			
Race						
Black						
White	0.77	0.60- 0.98	0.036			
Other	0.84	0.54- 1.29	0.423			
Histological type						
Leiomyosarcoma, NOS	Reference					
Epithelioid leiomyosarcoma	1.07	0.57-2.01	0.833			
Myxoid leiomyosarcoma	1.15	0.57-2.32	0.697			
Grade						
I-II	Reference					
III-IV	2.78	1.37- 5.68	0.005	3.17	1.55-6.51	0.002
Unknown	2.79	1.37- 5.71	0.005	2.61	1.27-5.40	0.009
Size						
≤50	Reference					
51-100	0.84	0.53- 1.36	0.483			
≥100	1.14	0.73- 1.77	0.572			
T-Stage.						
T1-T2	Reference					
T3-T4	1.56	1.22- 2.00	<0.001	1.69	1.31 − 2.17	<0.001
N-stage						
N0	Reference					
N1	1.20	0.84-1.70	0.31			
Surgery						
No	Reference					
Yes	0.49	0.36- 0.66	<0.001	0.49	0.36 − 0.68	<0.001
Radiation						
No	Reference					
Yes	1.04	0.77- 1.42	0.795			
Chemotherapy						
No/Unknown	Reference					
Yes	0.66	0.51- 0.85	0.001	0.61	0.47 − 0.79	<0.001

### Development and validation of prognostic nomogram

**Figure 4:**
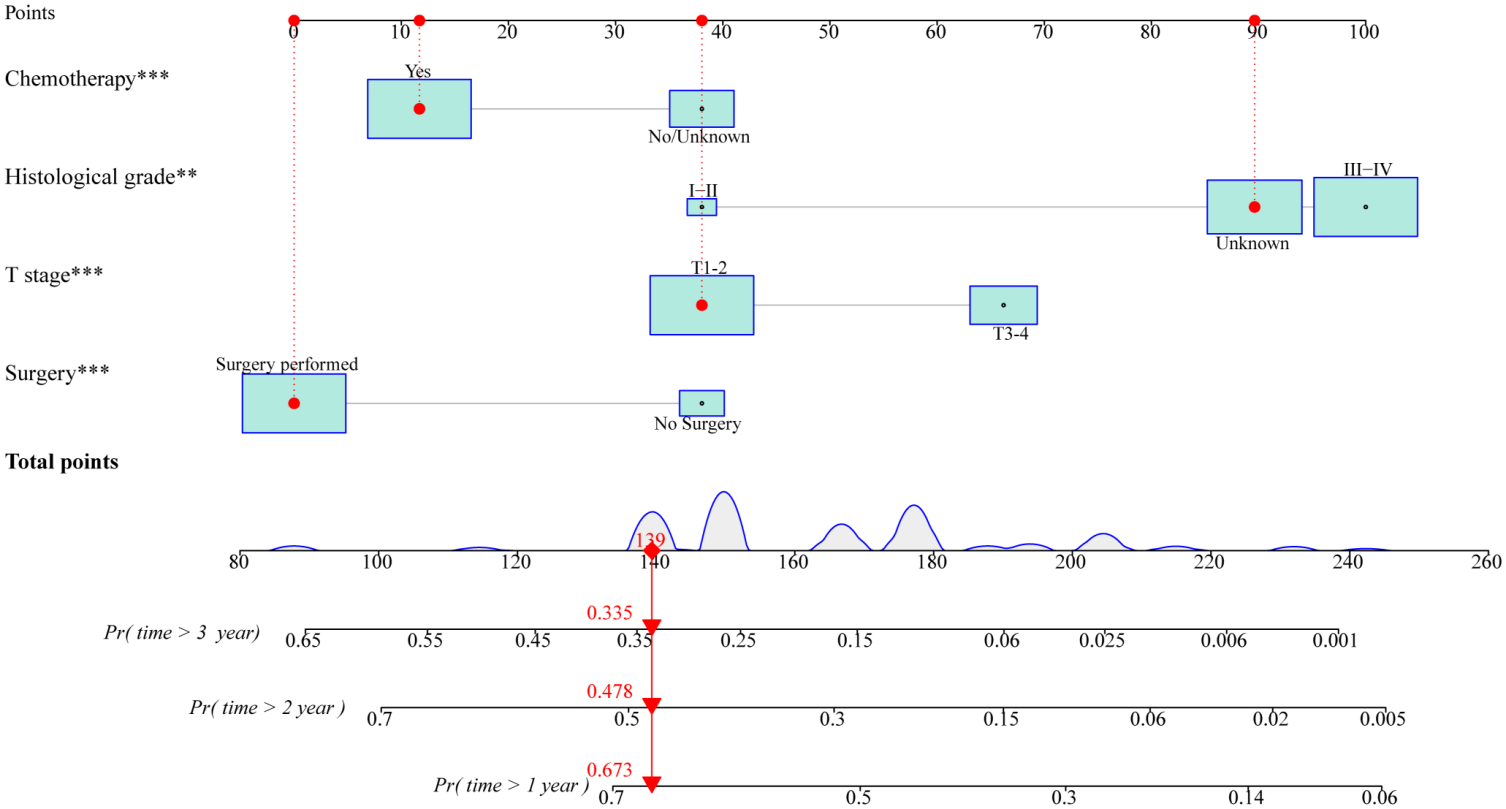
A prognostic nomogram for predicting OS at 1-, 2-, and 3-year in uLMS patients with distant metastases based on independent prognostic factors (histological grade, T stage, surgery, and chemotherapy). The size of the box reflected the distribution of the variables, with a larger size indicating a higher proportion. Each level of these variables was assigned a specific point on the scale, and a total point could be obtained for the individual patients by summing each point form the above four independent prognostic factors. A line was drawn vertically downward from the total points scale to the survival axes to determine the probability of 1-, 2-, and 3-year OS.

**Figure 5:**
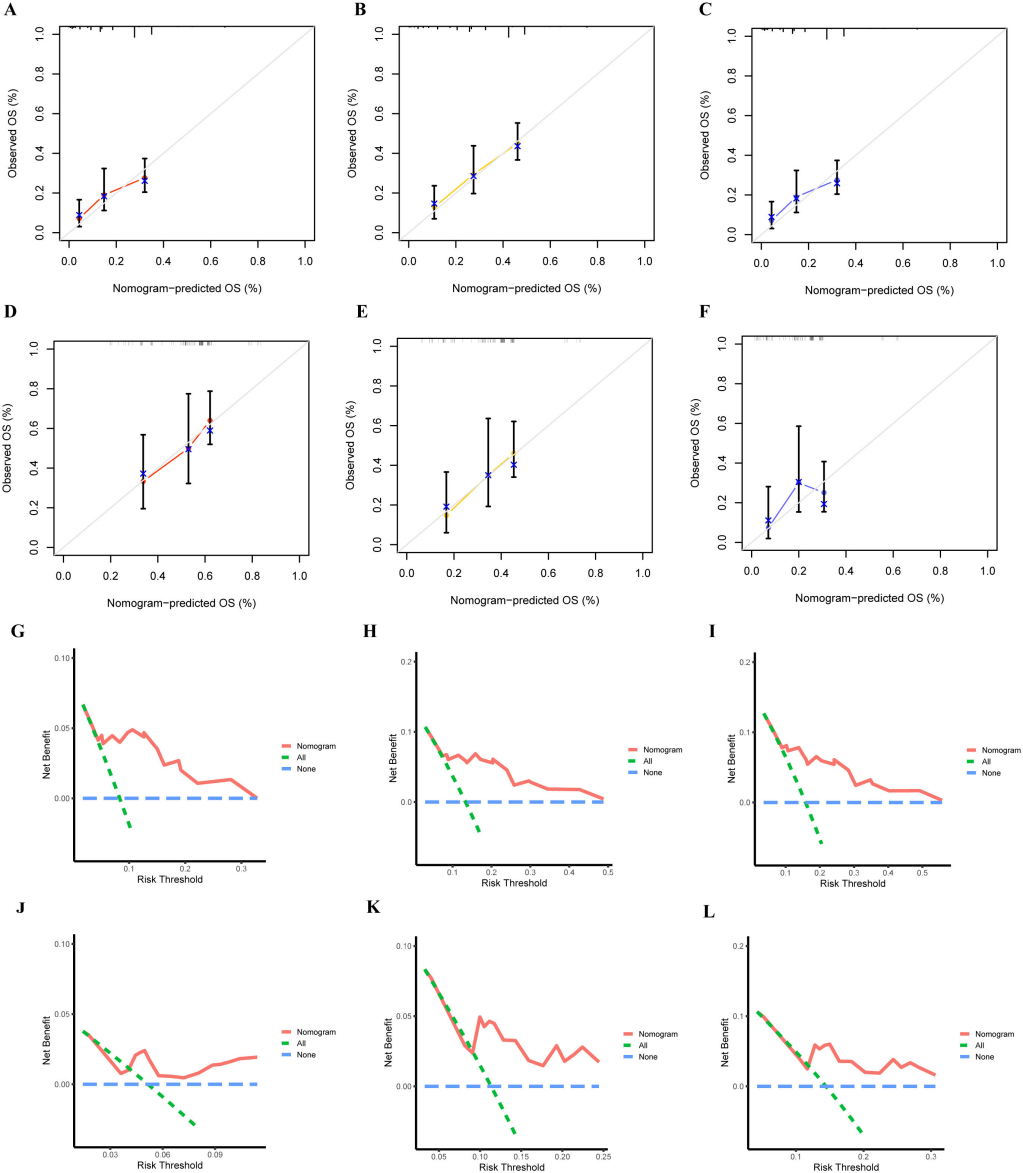
The accuracy of the prognostic nomogram was assessed by plotting the nomogram’s 1-,2- and 3-year prediction calibration curves in training **(A–C)** and validation **(D, F)** cohorts; DCA curves of 1- **(G)**, 2- **(H)**, and 3-year **(I)** OS in the training cohort and 1- **(J)**, 2- **(K)**, and 3-year **(L)** OS in the validation cohort.

**Figure 6:**
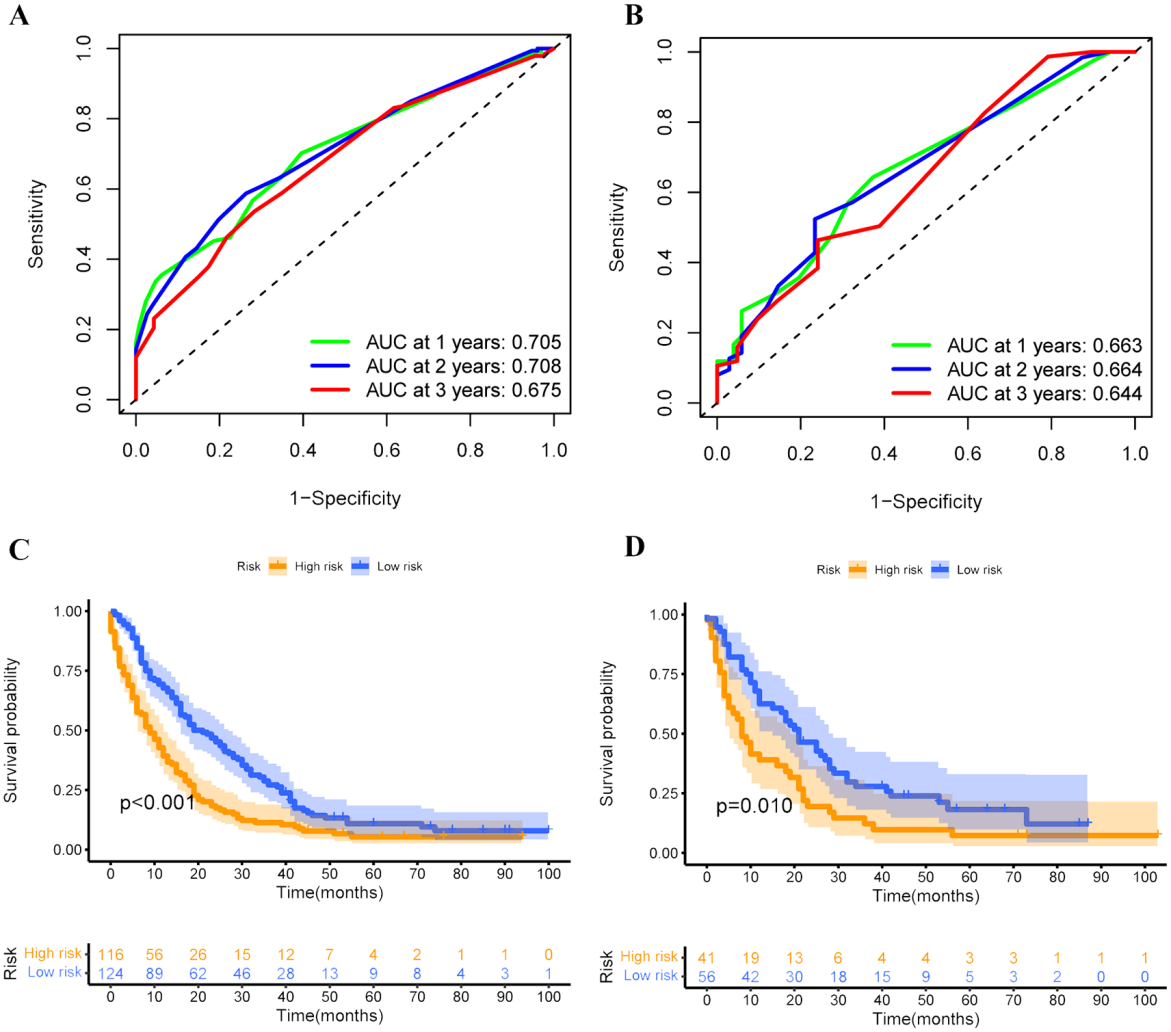
Time-dependent ROC curves were employed to evaluate the discriminative ability of 1-, 2-, and 3-year survival prediction in uLMS patients having distant metastases in both training **(A)** and validation **(B)** cohorts; Kaplan-Meier survival curves depicting the survival outcomes of high and low-risk groups in both training **(C)** and validation **(D)** cohorts.

Based on these independent prognostic variables selected by multivariate analysis, we constructed a prognostic nomogram to patients’ overall survival (OS) named “Metastatic uLMS Prognostic Index” in the training cohort and subsequently validated it in the validation cohort (**Figure 4**). A total score was derived by summing the point of individual risk factors, which could be applied to predict patients’ OS at 1-, 2-, and 3-year. Survival calibration plots demonstrated that the nomogram-predicted survival probabilities exhibited excellent consistency with the actual OS at 1-, 2-, and 3-year in both training (**Figures 5A–C**) and validation cohorts (**Figures 5D–F**). Moreover, DCA revealed large positive net gains in predictive nomogram across various threshold probabilities at different points in time (**Figures 5G–L**). Additionally, the time-dependent ROC curves at 1-, 2-, and 3-year further validated the nomogram’s discriminative capability in predicting the OS of patients with distant metastases, with corresponding AUCs of 0.835, 0.747, and 0.758 in the training cohort as well as 0.792, 0.831, and 0.786 in the validation cohort (**Figures 6A, B**). Then, we calculated the total score of every uLMS patient having distant metastases, and assigned them to low-risk and high-risk groups based on a median score. The Kaplan-Meier survival analysis showed that patients at high risk had a worse prognosis than those who were at low risk in both training and validation cohorts, underscoring the prognostic nomogram was promising to be an effective tool in risk stratification for such patients (**Figures 6C, D**).

### Validation of the prognostic nomogram in an expanded cohort

**Figure 7:**
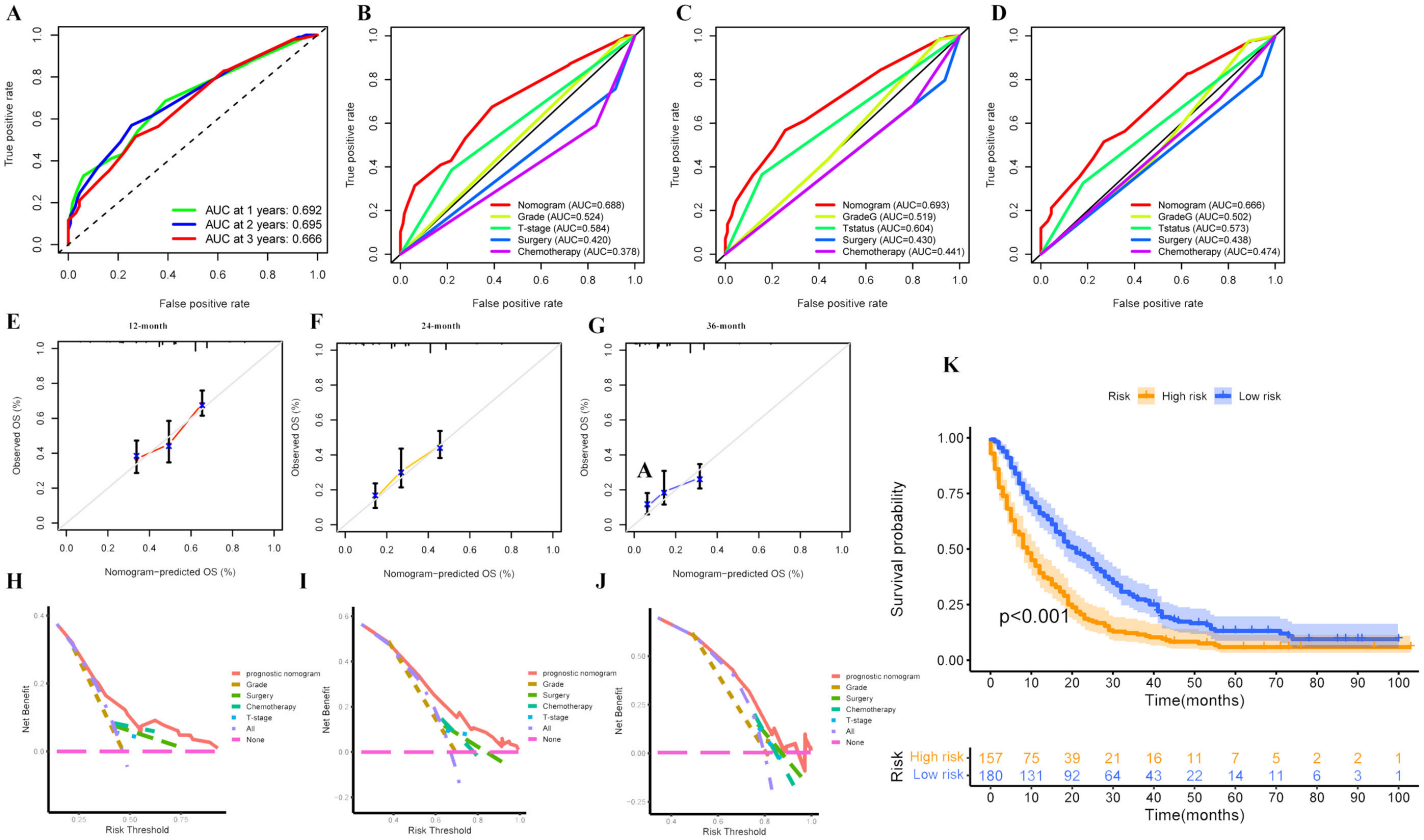
**(A)** Time-dependent ROC curves at 1-, 2-, and 3-year to evaluate the validity of the prognostic nomogram in the expanded cohort; The discrimination between the prognostic nomogram and independent prognostic variables (histological grade, T stage, surgery, and chemotherapy) for predicting survival at 1- **(B)**, 2- **(C)**, and 3-year **(D)** was compared by ROC curves in the expand cohort; Calibration curves were employed to evaluate the accuracy of the prognostic nomogram for predicting survival at 1- **(E)**, 2- **(F)**, and 3-year **(G)** in the expand cohort; DCA curves of the prognostic nomogram and independent prognostic variables at 1- **(H)**, 2- **(I)**, and 3-year **(J)** in the expanded cohort. **(K)** Kaplan-Meier survival curves depicting the survival outcomes of high and low-risk groups in the expanded cohort.

Besides, we re-screened and selected 337 eligible patients with complete histological type, T stage, surgery, and chemotherapy data to create an expanded cohort to further validate the prognostic nomogram’s predictive performance. The ROC analysis indicated that the values of AUC for OS prediction at 1-, 2-, and 3-year were 0.760, 0.738, and 0.738, respectively, superior prediction accuracy compared to four independent prognostic factors (**Figures 7A–D**). The calibration curves demonstrated favorable alignment between predicted and observational values (**Figures 7E–G**), while the DCA results suggested the positive net gain in predictive nomogram was greater than that of any single independent prognostic parameter across various threshold probabilities at different points in time, indicating this nomogram could be an effective clinical tool for predicting OS in uLMS patients with distant metastases (**Figures 7H–J**). Furthermore, the Kaplan-Meier survival analysis indicated that individuals categorized into the low-risk group exhibited significantly greater survival probabilities than those in the high-risk group (**Figure 7K**). In conclusion, the aforementioned findings underscored the excellent performance of the prognostic nomogram we developed in predicting the prognosis of patients with uLMS and distant metastases.

## Discussion

uLMS arising from the myometrium is a rare tumor, accounting for approximately 1% of all uterine malignancies (4). Compared to other types of uterine cancers, uLMS is an aggressive tumor correlated with a high risk of recurrence regardless of stage at diagnosis, making up almost 70% of all uterine sarcomas and a significant proportion of uterine cancer fatalities (4, 19). A retrospective analysis revealed that uterine sarcomas have a poor prognosis, with uLMS being the worst prognostic histological variant (20). The positioning of uLMSs in the myometrium promoted vascular invasion and the formation of distant metastases at an early stage of the disease (21). The lung seemed to be the most frequent metastatic site of uLMS, with bone, cranial/intracranial, and skin and soft-tissue metastases also relatively common (22). Although the majority of cases (60%) were diagnosed at an early stage, the five-year survival rate ranged from 25% to 76%. Conversely, for patients with metastases at initial diagnosis, survival rates dropped to approximately 10%-15% (23). The significance of early identification for synchronous distant metastases in uLMS patients and individualized survival prediction of those with distant metastases are self-evident in optimizing medical decision-making. To our knowledge, this is the first metastases model-based analysis to identify high-risk patients with u-LMSs prone to distant metastases, and to provide precise survival predictions for those affected by distant metastases, which potentially may assist clinicians in developing individualized treatment for this rare tumor.

Recently, there were several studies have focused on distant metastases in u-LMSs, but most of them are primarily at the molecular level. They investigated the gene expression patterns of primary and metastatic lesions, and they identified genes that were overexpressed in each type of lesion. A novel orthotopic and metastatic model derived from uterine sarcoma tissue in KSN nude mice unveiled differential gene expression associated with cell proliferation and migration between orthotopic tumors with high and low metastatic potentials, including TNNT1, COL1A2, and ZIC1 (24). Additionally, Kodama M et al. uncovered a novel mechanism driving ULMS tumorigenesis and metastases, pinpointing ZNF217 and NRDC as potential targets for ULMS therapy (25). Nevertheless, it is crucial to acknowledge that these studies often suffered from small sample sizes and were single-center investigations lacking robust validation. As a result, the identified biomarkers remain impractical for immediate clinical application, hampering their utility in clinical management.

In this study, we initially integrated extensive clinicopathological characteristics of uLMS from the SEER database, identifying seven significant predictors for distant metastases in uLMS patients namely, race, histological grade, T stage, N stage, tumor size, surgery, and chemotherapy. The risk predictive nomogram was constructed based on these predictors presented good discrimination and calibration in both training and validation cohorts. Interestingly, it was unexpected that patients receiving chemotherapy were more likely to develop metastatic disease. A growing body of preclinical evidence has revealed scenarios in which chemotherapy can trigger intra-tumoral and systemic alterations, paradoxically enhancing tumor cell survival and proliferation, thereby leading to the spread of cancer cells to distant organs. At the site of the primary tumor, chemotherapy-induced selection operated within the framework of intratumoral heterogeneity, affecting specific clones or cellular subsets, such as cancer stem cells (CSCs), which possessed inherent drug-resistant properties and enabled them to evade the effects of chemotherapy (26, 27). Chemotherapy was also considered to generate CSCs by acting EMT program, thereby permitting CSC-mediated clinical relapse (28). In addition to its direct effects on tumor cells, chemotherapy could trigger host-mediated pro-metastatic alterations through the systemic release of cytokines and chemokines, which might in turn promote the expansion of the subset of CSCs responsible for tumor relapse and the generation of metastasis-receptive niches by recruiting both tumor cells and supportive stromal cells at distant sites (29). More studies in this respect are needed in the future.

In recurrent or metastatic disease, anthracycline-based therapies such as doxorubicin plus ifosfamide, doxorubicin plus olaratumab, and gemcitabine plus docetaxel remain the mainstay of the management (2). Although uLMS patients receiving chemotherapy dramatically developed metastatic disease, our results showed that the absence of chemotherapy had a significant negative impact on OS. Besides, we also found that surgery was an independent protective factor for the prognosis in uLMS patients with distant metastases, whereas higher histological grade and T stage were independent risk factors. Based on the results of Cox analyses, we established a prognostic nomogram to predict the survival of u-LMS patients with distant metastases. Although two previous studies had conducted reliable nomograms for predicting overall survival in patients with uLMS, it was notable that they were not specifically designed for metastatic disease (30, 31). In our study, we established for the first time a nomogram specifically designed to predict the prognosis of u-LMS patients with distant metastases. The newly proposed prognostic nomogram demonstrated satisfactory predictive efficiency and practical value, exhibiting superior discriminative ability compared to individual independent prognostic factors.

Although our study was conducted at the population level with a significant number of cases, several limitations must be acknowledged. Firstly, due to the retrospective design, the study was susceptible to inherent selection biases. Secondly, the rarity of uLMS posed challenges in obtaining external data, leading to the lack of validation of the model by an external cohort. Thirdly, the limited parameters recorded in the SEER database prevented consideration of other clinical factors and biomarkers not included, such as immunotherapy, targeted therapy, postoperative complications, and gene expression characteristics, which might influence outcomes.

## Conclusions

In summary, our study identified race, histological grade, T stage, N stage, tumor size, surgery, and chemotherapy as independent risk factors for distant metastases in uLMS. Additionally, surgery, chemotherapy, histological grade, and T stage were identified as independent prognostic factors for uLMS patients with distant metastases. Based on these findings, we developed well-validated risk and prognostic nomograms to accurately estimate the likelihood of metastases and predict the prognosis of those with distant metastases, offering valuable guidance for individualized clinical management for these patients.

## Data Availability

The original contributions presented in the study are included in the article/**Supplementary Material**. Further inquiries can be directed to the corresponding authors.
