# Supplementary material for: Individualized evaluation of risk and prognosis in uterine leiomyosarcoma patients with synchronous distant metastases: a real-world retrospective study
Source: Front Oncol. 2024 Sep 25;14:1417226. doi: 10.3389/fonc.2024.1417226 (PMC11461169; doi:10.3389/fonc.2024.1417226)
Supplement: Supplementary file 1 [file DataSheet1.pdf]

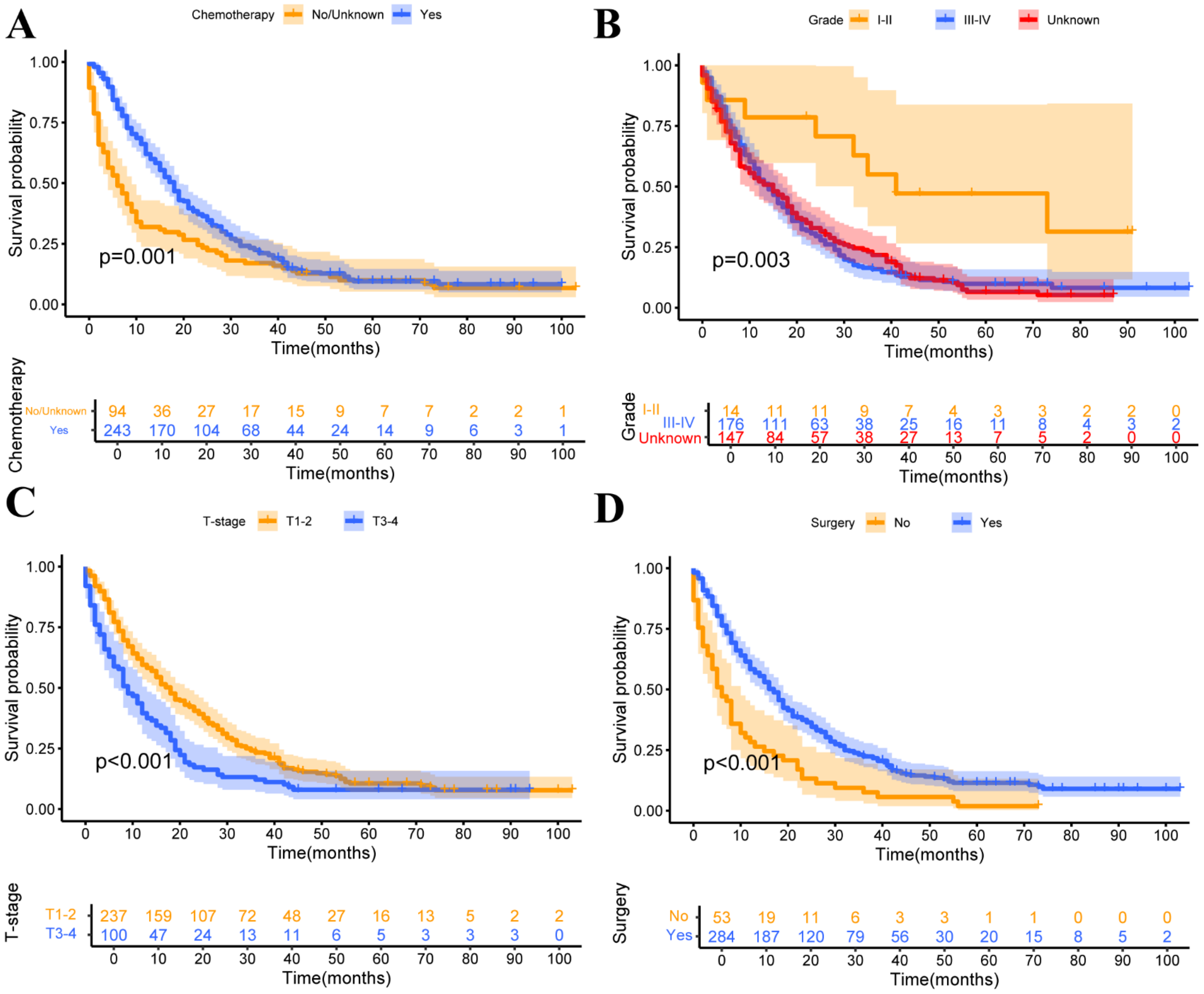

Figure S1. The Kaplan–Meier survival curves showed the different effects of parameters on the OS according to the results of the multivariable analysis. **A.** Chemotherapy ($P = 0.001$); **B** Grade ($P = 0.003$); **C** T-stage ($P < 0.001$); **D** Surgery ($P < 0.0001$).
